# Efficacy of Nusinersen Treatment in Type 1, 2, and 3 Spinal Muscular Atrophy: Real-World Data from a Single-Center Study

**DOI:** 10.3390/neurolint16060096

**Published:** 2024-10-29

**Authors:** Anna Lemska, Piotr Ruminski, Jakub Szymarek, Sylwia Studzinska, Maria Mazurkiewicz-Beldzinska

**Affiliations:** 1 Department of Developmental Neurology, Medical University of Gdansk, 80-952 Gdansk, Poland; 2 Faculty of Chemistry, Nicolaus Copernicus University, 87-100 Torun, Poland

**Keywords:** spinal muscular atrophy, nusinersen, motor function, real-world data

## Abstract

Background: Spinal muscular atrophy (SMA) is an inherited neuromuscular disease characterized by progressive muscle weakness and atrophy due to the absence of the survival motor neuron 1 (*SMN1*) gene. SMA is classified into types 0 through 4 based on the age of symptom onset and the severity of motor function decline. Recent advances in SMA treatment, including nusinersen, onasemnogene abeparvovec, and risdiplam, have significantly improved the prognosis of SMA patients. This study evaluated the safety and efficacy of nusinersen in pediatric patients with SMA types 1, 2, and 3 in a real-world clinical setting. Methods: This prospective observational single-center study assessed the treatment effects of nusinersen in 23 pediatric patients with genetically confirmed SMA over a 22-month observation period. All the participants received intrathecal loading doses of 12 mg of nusinersen on days 1, 14, 28, and 63, followed by maintenance doses every four months. Functional assessments were conducted using the CHOP-INTEND scale. Data were collected during routine patient visits, including clinical laboratory tests and vital sign parameters, and adverse events were recorded. The inclusion criteria were defined by the national reimbursement program for nusinersen treatment in Poland. Results: Initially, 37 patients ranging from 1 month old to 18 years old were included, but 23 were ultimately observed due to changes in treatment regimens or assessment scales. The patients showed significantly improved CHOP-INTEND scores over the 22-month period. At 6 months, the average increase was 4.2 points, continuing to 17.8 points at 22 months. By the end of the study, 100% of patients showed either stabilization or improvement, with significant clinical improvements observed in several patients. Nusinersen was generally well-tolerated, with post-lumbar puncture headache and lower back pain being the most common adverse events. Conclusions: Nusinersen treatment significantly enhances motor function in pediatric patients with SMA types 1, 2, and 3. This study demonstrates the importance of early and sustained treatment, with most patients showing the continuous improvement or stabilization of motor function. These findings support the use of nusinersen as an effective therapy for SMA; however, further research is needed to understand the long-term outcomes and optimize treatment strategies.

## 1. Introduction

Spinal muscular atrophy (SMA) is an inherited neuromuscular disease marked by progressive muscle weakness and atrophy, and it is caused by the absence of the *survival motor neuron 1* (*SMN1*) gene. SMA is classified into types 0 through 4 based on the age of symptom onset and the severity of motor function decline. The severity of SMA is influenced by the number of copies of the *SMN2* gene, which partially compensates for the lack of *SMN1* by producing some functional SMN protein. More *SMN2* copies typically result in milder symptoms. SMA affects people of all ages and has a wide range of severity, impacting individuals’ motor skills and overall quality of life [1,2]:

SMA Type 0: This type has a prenatal onset, with mothers often noticing decreased fetal movement late in pregnancy. Infants are born with severe weakness, hypotonia, facial diplegia, and sometimes congenital heart defects or arthrogryposis. These infants achieve no motor milestones and usually die from respiratory failure, often within the first month. Typically, they have only one copy of the *SMN2* gene.

SMA Type 1: Also known as Werdnig–Hoffmann disease, this type presents before six months of age. Infants initially appear normal but soon develop severe, symmetric flaccid paralysis and never sit unsupported. Respiratory muscle weakness leads to respiratory failure, and most infants die before the age of two. Patients generally have two or three copies of the *SMN2* gene

SMA Type 2: This intermediate form presents between 3 and 15 months of age. Children are able to sit but never stand or walk independently. Common symptoms include proximal muscle weakness, areflexia, scoliosis, and respiratory insufficiency, leading to a variable life expectancy. Most patients have three copies of the *SMN2* gene.

SMA Type 3: Known as Kugelberg–Welander disease, this type presents between 18 months of age and adulthood. Patients achieve independent ambulation but may lose it over time, becoming wheelchair-dependent. They have proximal weakness, particularly in the legs, but usually maintain a normal lifespan. This type is associated with three or four copies of the *SMN2* gene.

SMA Type 4: The late-onset form, accounting for fewer than five percent of cases, typically begins after the age of 30. Patients achieve all motor milestones, maintain ambulation throughout life, and have a normal lifespan. They usually have four to eight copies of the *SMN2* gene [3,4,5].

SMA occurs in approximately 5 to 13 out of every 100,000 live births. The frequency of carriers of the *SMN1* gene mutation ranges from 1:100 to 1:45, varying significantly across different ethnic groups. SMA has long been regarded as the leading genetic cause of death in infants [1,6,7].

The treatment options for SMA have advanced with the introduction of three key medications: nusinersen, onasemnogene abeparvovec, and risdiplam. Nusinersen (Spinraza^®^) works by altering the splicing of the *SMN2* gene to boost the production of the full-length SMN protein. It has led to significant motor function improvements in children with both early- and later-onset SMA and is approved for use regardless of the patient’s age, *SMN2* copy number, or motor ability. Onasemnogene abeparvovec (Zolgensma^®^) is a gene therapy that replaces the faulty *SMN1* gene, offering a one-time treatment to prevent disease progression. Risdiplam (Evrysdi^®^) is an oral medication that enhances SMN protein production by modifying the *SMN2* splicing. These treatments have transformed the outlook for patients with SMA by significantly enhancing their motor function and their quality of life [8,9,10,11]. Nonetheless, more research is needed to fully understand the long-term effects of these therapies, and to fine-tune the treatment strategies and evaluation methods. Reports on the effectiveness of these treatments based on real-world data are particularly interesting.

## 2. Materials and Methods

In this prospective observational single-center study, we assessed the safety and treatment effects of nusinersen in 23 pediatric patients with SMA over a 22-month observation period. All participants had a genetically confirmed diagnosis and received treatment between January 2019 and July 2024. A current genetic report, including the *SMN2* copy number, was required before the initiation of treatment. In the genetic study, the patients were found to have either two copies or three copies of the *SMN2* gene. Therefore, the study included patients with types 1, 2, and 3 of SMA. Patients with SMA type 0 were not included in our study, as per the current treatment program in Poland. Patients with this type are not eligible for nusinersen therapy due to data suggesting limited effectiveness of the treatment in this group. Instead, SMA type 0 patients receive palliative care [12,13,14]. The average age at the start of therapy was 31.24 months for patients with two copies, 31.09 months for patients with three copies, and 31.09 months for all patients combined. Dividing the patients into specific groups, those with two copies of the *SMN2* gene started treatment between 1 and 98 months of age, while patients with three copies began treatment between 18 and 86 months of age. Data were collected during routine patient visits, and the items for data collection were aligned with the international consensus guidelines for SMA registries. All patients provided written informed consent. The inclusion criteria were defined by the national reimbursement program for nusinersen treatment in Poland [13]. The patients had no contraindications for lumbar puncture or any inability to undergo this procedure. Table 1 summarizes the baseline characteristics of all patients:

### 2.1. Treatment Schedule

All patients received intrathecal loading doses of 12 mg of nusinersen on days 1, 14, 28, and 63, followed by maintenance doses every four months. Intrathecal injections were performed without imaging guidance. Based on patient preference, local anesthesia (5% lidocaine cream) or sedation was offered. Patients were monitored for at least 8 h after each procedure in case any early adverse events occurred. Clinical laboratory tests were performed before each injection, including those that measured liver enzymes (AST, ALT, and GGT), creatinine, creatine kinase (CK), morphology, and coagulogram. Vital sign parameters, including blood pressure, heart rate, and peripheral oxygen saturation, were collected throughout the entire 8 h observation. Adverse events were recorded from baseline until the last day of observation.

### 2.2. Functional Assessment

At each visit, the same physiotherapist evaluated the patients using the Children’s Hospital of Philadelphia Infant Test of Neuromuscular Disorders (CHOP-INTEND) scale. This tool is specifically designed to assess motor function in infants with neuromuscular disorders, particularly SMA. The scale comprises 16 tasks that measure spontaneous movement, muscle strength, and reflexes, with each task scored between 0 and 4, resulting in a total score ranging from 0 to 64. The CHOP-INTEND test is widely used to establish baseline motor function and track progress over time, offering valuable insights into treatment effectiveness [15,16]. In our study, the CHOP-INTEND test was used to assess young patients up to 24 months of age as well as non-sitting patients. This method facilitated a thorough evaluation of motor function in the selected patient groups, offering important insights into their treatment responses. To maintain consistency, only patients who had undergone CHOP-INTEND assessments from the start of treatment up to the data cutoff were included. The assessments were carried out consistently between 10 a.m. and 1 p.m., upon ward admission, approximately one hour after feeding, ensuring that the children were in their best possible state for evaluation. The tests were performed on a firm mat, with the children wearing minimal clothing or just a diaper. Every effort was made to complete the assessments in one session, with parents stepping in briefly to comfort the child if needed. The evaluation was conducted in accordance with the recommendations [15]. For each evaluated patient, we also record the infant’s emotional state during the specific attempt using the Brazelton Neonatal Behavior Assessment Scale (NBAS), a behavioral assessment tool for newborns [15,16]. However, this was not the subject of evaluation in the study being described.

## 3. Results

Initially, the study included 37 patients ranging in age from 1 month old to 18 years old, all of whom were diagnosed with SMA and treated with nusinersen. However, ultimately, only 23 patients were included in the final observation. The remaining patients were excluded primarily due to changes in their treatment regimens, with the patients’ treatment changing to risdiplam or onasemnogene abeparvovec. None of the patients received combination therapy with nusinersen and risdiplam or onasemnogene abeparvovec. None of the patients decided to discontinue the treatment, and, consequently, no one withdrew from the observation period. Additionally, some patients were excluded because they were assessed using a different scale, specifically the Hammersmith Functional Motor Scale Expanded (HFMSE) rather than the CHOP-INTEND scale used in this study. Patients who had been observed for less than 22 months were also excluded. This selection process aimed to ensure that a homogeneous group of patients was included in this study for more consistent and reliable data analysis.

The first patient included in the analysis received their first dose of medication in January 2019, and the last patient began treatment in August 2022. Each patient was observed for a minimum of 22 months. After this period, the collected clinical data, including physiotherapy scale assessments and adverse events, were analyzed.

### 3.1. Safety

The intrathecal administration of nusinersen was generally tolerated well. Post-lumbar puncture headache was reported in 10 patients, occurring a total of 13 times out of 138 punctures (9%). Four patients experienced lower back pain within 24 h following the intrathecal injection. None of the patients exhibited the clinical symptoms of communicating hydrocephalus. The laboratory tests to check for adverse events were all unremarkable.

One of the patients passed away just before the administration of the ninth dose of nusinersen. According to the information obtained, the death was caused by respiratory failure due to severe pneumonia. The death was not classified as a complication of nusinersen administration. Given that the patient had completed the 22-month observation period, he was included in the study.

From the first visit to the 22-month visit, this patient’s CHOP-INTEND score significantly increased following the administration of the medication. However, this case highlights the challenging and variable progression of spinal muscular atrophy.

### 3.2. Treatment Effects

**Figure 1:**
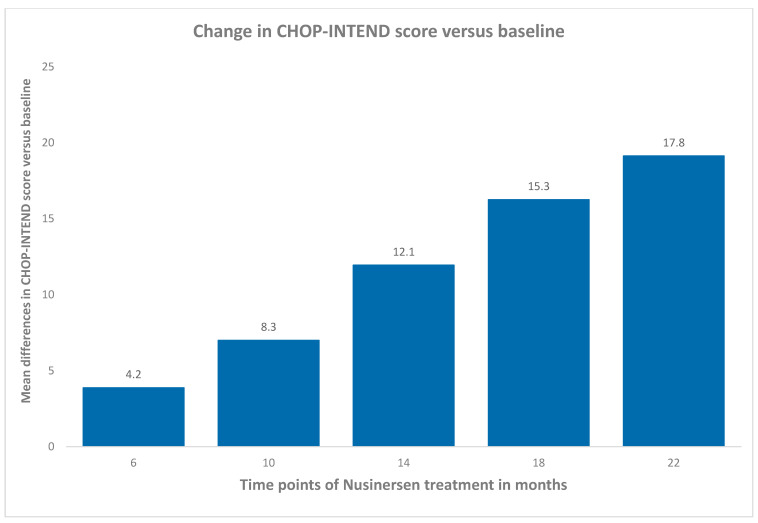
Change in CHOP-INTEND score versus baseline.

Figure 1 illustrates the average change in the CHOP-INTEND scores over time for the patients receiving nusinersen treatment for up to 22 months. At 6 months, the average increase in the CHOP-INTEND score was 4.2 points. This improvement continued steadily, with an 8.3-point increase at 10 months, a 12.1-point increase at 14 months, a 15.3-point increase at 18 months, and the highest increase of 17.8 points at 22 months. These results indicate that the most significant therapeutic benefits of nusinersen are observed over the course of treatment, as patients consistently show steady and substantial improvements in their motor function scores, even though some patients did not show improvement at the beginning of the observation period.

**Figure 2:**
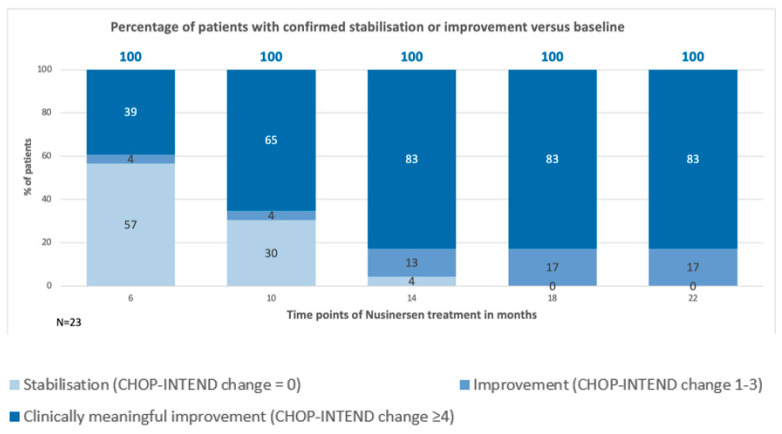
Percentage of patients with confirmed stabilization or improvement versus baseline.

Figure 2 shows the percentage of patients whose condition was either stable or improved compared to their baseline values up to 22 months. At 6 months, 100% of paztients showed either stabilization or improvement, with 39% showing clinically meaningful improvement, 57% showing stabilization, and 4% showing improvement. This trend continued, with 100% of patients showing either stabilization or improvement at 10, 14, 18, and 22 months. Specifically, at 10 months, 65% showed clinically meaningful improvement, 30% showed stabilization, and 4% showed improvement. By 14 months, 83% of patients improved more than 3 points in CHOP-INTEND, 13% improved 1 or 2 points in CHOP-INTEND, and 4% showed stabilization. At 18 and 22 months, 83% of patients significantly improved, with a notable 17% improvement. Stabilization was defined as no change in the CHOP-INTEND score. Improvement was defined as an increase in the score by 1 to 3 points. Patients who showed an improvement of more than 4 points in the CHOP-INTEND scale were classified as having significant clinical improvement.

**Figure 3:**
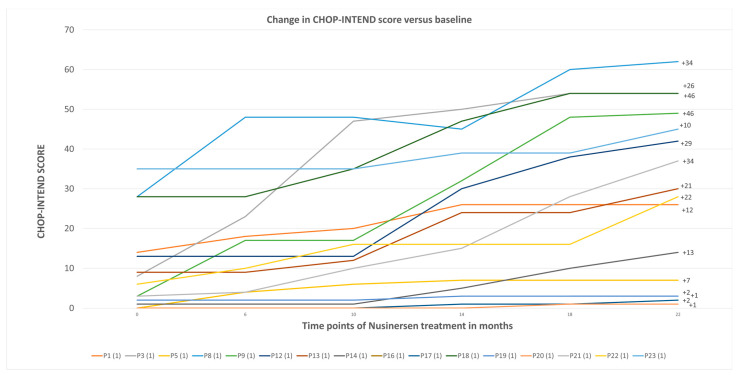
Change in CHOP-INTEND score versus baseline.

Figure 3 depicts the change in CHOP-INTEND scores over time for individual patients receiving nusinersen treatment for up to 22 months. The data reveal several key insights into the efficacy of the treatment and the variability in patient responses. Overall, there is a clear improvement in the CHOP-INTEND scores for most patients over the 22-month period, indicating that nusinersen treatment generally leads to enhanced motor function in patients with SMA.

The study included two patients with SMA type 3; five patients with SMA type 2; and sixteen patients with SMA type 1. This distribution of patients likely reflects the study design, as we planned to analyze only those patients who were assessed using the CHOP-INTEND scale. This scale is primarily used to evaluate patients with SMA type 1 or small children. The patients with SMA type 3 were assessed with this scale up to 24 months of age, and, after reaching 2 years old, they were evaluated using the HFMSE scale. In the case of patients with SMA type 2, the decision to use the CHOP-INTEND scale was based on their functional status; non-sitters were evaluated.

The analysis revealed that patients with SMA type 3 achieved the highest scores on the CHOP-INTEND scale, with respective scores of 64 and 58 out of a maximum of 64 points. These patients had very high baseline assessments in this scale, and, due to reaching the maximum possible score, they were subsequently evaluated using another physiotherapeutic scale. Upon reviewing the results, it was observed that the greatest improvement was seen in patients with SMA type 1, particularly those who started treatment at a very early stage, within the first months of life following diagnosis. These patients showed an improvement of up to 46 points on the CHOP-INTEND scale. However, one patient, despite receiving early treatment in the first months of life, did not show such a significant improvement in CHOP-INTEND scores. This may be attributed to the patient’s poor functional status before treatment initiation.

**Figure 4:**
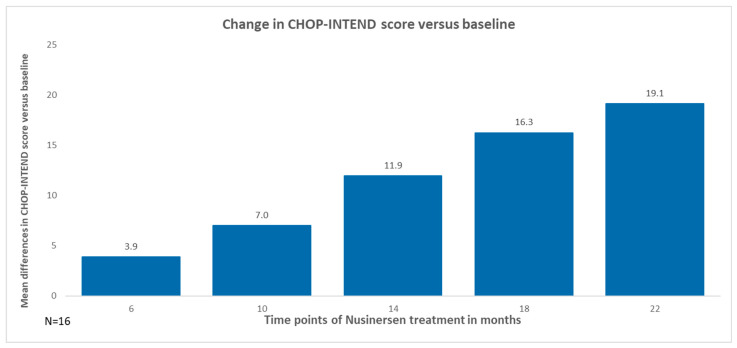
Change in CHOP-INTEND score versus baseline (SMA type 1).

**Figure 5:**
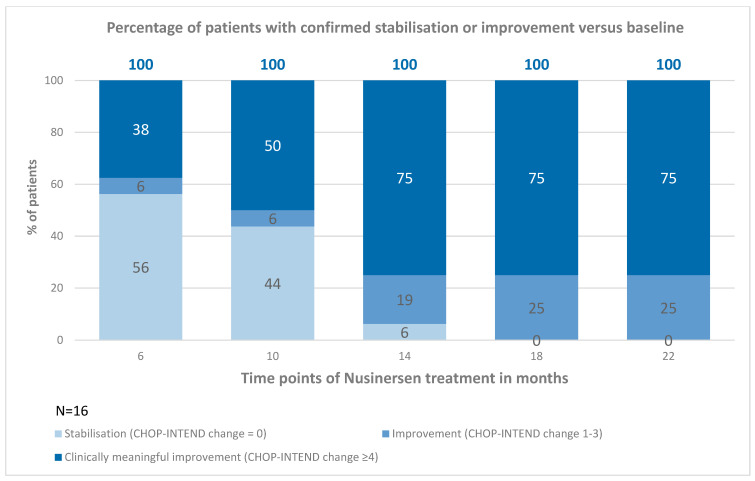
Percentage of patients with confirmed stabilization or improvement versus baseline (SMA type 1).

In patients with SMA type 1, the improvement on the CHOP-INTEND scale after 6 months of observation was 3.9 points; after 10 months, 7 points; after 14 months, 11.9 points; after 18 months, 16.3 points; and, after 22 months, 19.1 points, as illustrated in Figure 4. The percentage of patients who improved after 22 months of observation compared to baseline was 100%, with 25% of them showing an improvement of 1 or 2 points, while the remaining patients showed an improvement of at least 3 points as illustrated in Figure 5.

**Figure 6:**
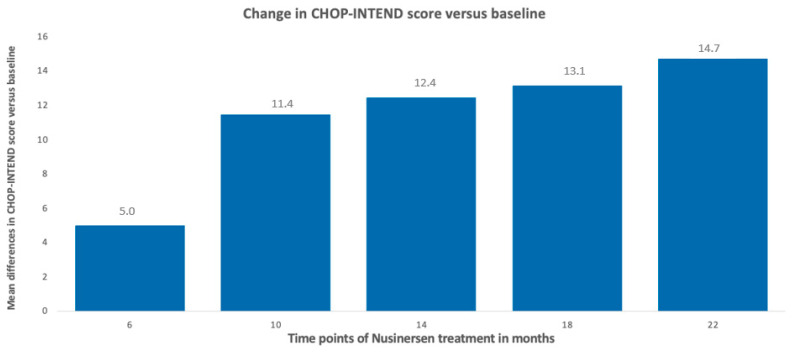
Change in CHOP-INTEND score versus baseline (SMA type 2 and type 3).

**Figure 7:**
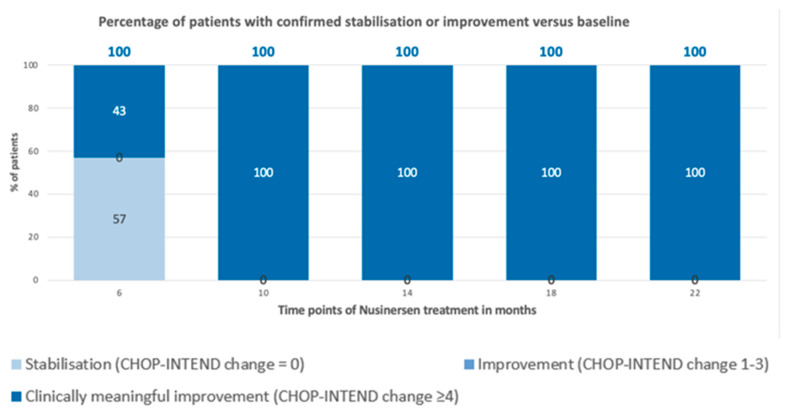
Percentage of patients with confirmed stabilization or improvement versus baseline (SMA type 2 and 3).

Patients with SMA types 2 and 3 were analyzed together due to the small sample size. After 6 months of treatment, the average improvement on the scale was 5 points; after 10 months, 11.4 points; after 14 months, 12.4 points; after 18 months, 13.1 points; and, after 22 months, the improvement averaged 14.7 points. It was noted that, from 10 months of observation, 100% of patients showed clinically meaningful improvement (a minimum of 3 points of improvement on the CHOP-INTEND scale); these data are illustrated in Figure 6 and Figure 7.

## 4. Discussion

Natural history, as evidenced from various studies, suggests that the natural course of SMA is characterized by a steady decline in motor function in all patients, regardless of the specific type of the disease. Over time, individuals with SMA experience worsening symptoms, including a gradual loss of muscle strength and mobility. It has been well-established that the earlier the onset of symptoms is, the more rapid and severe the disease progression tends to be. Patients who develop symptoms at a younger age, particularly in infancy, often show the fastest and most significant deterioration compared to those whose symptoms appear later in life. This highlights the importance of early intervention and treatment to slow the progression of the disease [17,18,19]. The development of three disease-modifying treatments has significantly transformed the management of SMA. The introduction of these drugs was preceded by a series of clinical trials on treatment efficacy that were all successful [8,20]. However, reports from everyday clinical practice, which are still relatively few, seem particularly interesting.

In our study, the group of patients included for observation may seem small. However, considering the prevalence of SMA in the population and the need to select a highly homogeneous group, our sample is representative of real clinical conditions. Furthermore, focusing on a carefully selected group of patients allows a more precise assessment of the treatment efficacy to be conducted and a better understanding of the outcomes in the context of everyday medical practice to be obtained.

The results of our study highlight the significant therapeutic benefits of nusinersen in improving motor function in patients with SMA, as evidenced by the increase in CHOP-INTEND scores over a 22-month period. At 6 months, patients exhibited an average increase of 4.2 points in their CHOP-INTEND scores, which improved progressively and had increased by an average of 17.8 points by 22 months. This consistent enhancement in motor function underscores the efficacy of nusinersen in the patient population, even though some individuals did not show an improvement at the beginning of the observation period. We believe that this is due to the initial condition of these patients. It was observed that patients who had worse baseline clinical assessments scored lower on the CHOP-INTEND scale. These were patients for whom treatment was initiated at a more advanced stage of the disease. However, it is important to note that, despite their advanced clinical state, these patients also benefited from the treatment. The benefit was not always measurable with the CHOP-INTEND scale. Attempts were made to assess these patients using the RULM scale, but the group was too small to achieve statistically significant results. Parents and caregivers reported improvements in bulbar functions, a greater resistance to infections, and a longer attention span during physiotherapy or school activities; some of these improvements are difficult to measure with the available scales. Therefore, it is valuable to continue observing patients in everyday clinical practice.

As a starting point for further research within this observation, the authors examined the results obtained using the HFMSE scale. The Hammersmith Functional Motor Scale Expanded (HFMSE) is, like CHOP-INTEND scale, a clinical assessment tool to evaluate the motor function of patients with neuromuscular disorders, particularly SMA. It consists of a series of tasks that measure physical abilities such as sitting, standing, walking, and other motor skills. The expanded version includes additional items to increase its sensitivity for detecting changes in motor function over time [21]. At the center where this observation was conducted, the HFMSE scale is used to evaluate patients with SMA types 2 and 3 after reaching the maximum score on the CHOP-INTEND scale, and patients who have reached 2 years of age. The analysis included 10 patients who had completed a 22-month observation period, all of whom were treated with nusinersen. Among these, six patients had SMA type 3, and four patients had SMA type 2. For all patients, the average change in HFMSE score compared to baseline after 22 months of observation was 7.1 points. The greatest changes were observed between the 6th and 10th months (an increase from 1.5 to 3.3 points) and between the 18th and 22nd months (an increase from 5.1 to 7.1 points).

Interestingly, after 22 months of observation, 90% of patients showed clinically significant improvement, defined as an increase of at least 3 points on the HFMSE scale. Additionally, 10% of patients showed an improvement of 1 or 2 points, while none of the patients experienced stabilization (no change in HFMSE score). Similar observations were made in the study by Hagenacker; clinically significant improvements, defined as an increase of 3 or more points in HFMSE scores, were observed in 35 out of 124 patients (28%) at 6 months, 33 out of 92 patients (35%) at 10 months, and 23 out of 57 patients (40%) at 14 months [22].

Taking all patients into account, regardless of the scale used for assessment, it was observed that those with higher baseline scores responded better to nusinersen compared to those with lower baseline scores. However, it is important to note that every patient benefited from the introduced treatment, regardless of their baseline scales’ scores.

Comparatively, our findings are consistent with other studies that have investigated the effects of nusinersen on motor function in SMA patients. For example, the pivotal ENDEAR study demonstrated that infants treated with nusinersen showed significant improvements in their motor milestones and motor function compared to a sham control group. In the ENDEAR study, a significant proportion of the treated infants achieved motor milestones that are rarely seen in untreated patients, such as sitting independently; some were even able to walk with assistance [23]. Similarly, the CHERISH study, which focused on later-onset SMA, found that the Hammersmith Functional Motor Scale Expanded (HFMSE) scores of children treated with nusinersen improved significantly compared to those in the control group [24].

Our study also showed that the CHOP-INTEND scores either stabilized or improved in 100% of patients up to 22 months, with a notable 17% showing significant clinical improvement at the end of the observation period. Stabilization was defined as no change in the CHOP-INTEND score, while improvement was an increase of 1 to 2 points, and significant clinical improvement was defined as an increase of more than 3 points. This high rate of stabilization and improvement aligns with findings from real-world data, such as those from the SMA REACH UK registry, which demonstrated similar outcomes in a broader clinical setting [25].

By analyzing the graph depicting each patient individually, we concluded that many patients show a significant improvement within the first 6 months of treatment. Several lines on the graph, representing different patients, indicate sharp increases in their CHOP-INTEND scores early in the treatment. This early improvement suggests that the action of nusinersen begins rapidly and enhances motor abilities. Additionally, some patients continue to show improvement beyond the 6-month mark, with a few showing a substantial improvement after 22 months. The scores of some patients stabilize, particularly after initial improvements. This indicates that, while not all patients experience continuous improvement, many can maintain their motor function levels without further decline. The highest gain, namely, +46 points, demonstrates a substantial recovery of motor function aided by nusinersen. Other notable improvements include gains of +34, +29, and +26 points, further highlighting the effectiveness of the treatment in enhancing these patients’ quality of life. In the study conducted by Lusakowska, nusinersen led to continuous functional improvement over a 30-month follow-up period and was well-tolerated by both adults and older children with a wide range of SMA severity. Similar to the population described in our study, patients across different ages and levels of disease progression benefited from the treatment, showing improvements in motor function and daily living activities.

Additionally, the safety profile of nusinersen remained favorable throughout the extended follow-up, with no new or unexpected adverse effects reported. This suggests that nusinersen is an effective and safe long-term treatment option for individuals with spinal muscular atrophy, regardless of the severity or stage of the disease [26]. Adverse events were reported in 9% of patients, the majority of which were mild. This is consistent with previous findings [27,28,29]. We observed that the most common side effects were headaches and back pain. To minimize these adverse events, proper positioning during lumbar puncture, adequate hydration before and after the procedure, and the careful monitoring of patient comfort can be effective strategies. Additionally, post-procedure rest and the use of mild analgesics may help in reducing the incidence and severity of these symptoms. Considering the growing number of patients who are being treated with nusinersen, it is crucial that we further investigate this area, which impacts the quality of life. This is especially important for pediatric patients, who may benefit the most. By focusing on the specific needs and challenges faced by younger patients, healthcare providers can work to reduce side effects.

Despite these positive outcomes, the scores of a few patients showed minimal or no change, indicating a need for further research to understand the factors that influence these varied responses to treatment. Patients who began nusinersen treatment at an earlier age demonstrated greater improvements in the CHOP-INTEND scale. Similar results were found by Aragon-Gawinska et al. [30] and Pane et al. [31], who reported significant improvements in motor function in younger patients. However, in the referenced studies, the observation period was shorter. This is expected, as untreated SMA is characterized by an early decline in motor function due to the rapid progression of motor neuron degeneration in the spinal cord. Therefore, incorporating a 5q SMA test into national newborn screening programs allows for the earlier initiation of SMA treatment, helping to prevent motor neuron degeneration, and the onset of severe disabilities. Moreover, the findings suggest that, while nusinersen is broadly effective, the characteristics of individual patients or other variables may impact its efficacy. The SMN2 gene is considered the key modifier of disease activity. However, the studies showed that SMA phenotypes were either inconsistent in terms of SMN2 copy numbers or that the gene had limited predictive power for individual outcomes. The severity of SMA cannot be fully explained only by the SMN2 copy number alone, as patients with the same number of copies may exhibit different SMA types. This indicates that other genetic or epigenetic factors likely contribute to the variability in SMA phenotypes [32,33,34,35].

The limitation of our study is undoubtedly the small patient cohort, which may impact the generalizability of the results. In the future, this issue could be addressed by conducting a multi-center study, which would enable a larger and more diverse group of patients. Additionally, extending the observation period could provide more comprehensive data and improve the reliability of the findings.

## 5. Conclusions

In conclusion, this study illustrates the positive impact of nusinersen on motor function in SMA patients, with most showing improvement over time. The degree of improvement varies among patients, with some achieving significant clinical gains. These findings highlight the importance of early and sustained treatment and the need for individualized approaches that maximize patient outcomes. Continued research and monitoring are essential in order to further refine treatment strategies and enhance the therapeutic benefits for all patients with SMA.

## Figures and Tables

**Table 1:** Characteristics of all patients.

Patient’s Demographics	*SMN2* 2 Copies	*SMN2* 3 Copies	All Patients
Sex (female–male)	6:11	3:3	9:14
Age of onset (average in months)	2.12	12.33	4.78
Age of diagnosis (average in months)	7.88	27.33	12.96
Age at start of therapy (average in months)	31.24	31.09	31.09

## Data Availability

The data are available upon reasonable request.
